# Dimethylacetamide-associated liver injury in workers: clinical patterns of latency, selective occurrence, and implications for occupational surveillance

**DOI:** 10.1093/joccuh/uiag043

**Published:** 2026-09-28

**Authors:** Jung-Min Sung, Young Joong Kang, Woncheol Lee

**Affiliations:** Occupational Safety and Health Research Institute, Korea Occupational Safety and Health Agency, Ulsan, Republic of Korea; Department of Occupational and Environmental Medicine, Ulsan Teo Clinic, Teo Occupational and Environmental Medicine Center, Ulsan, Republic of Korea, Republic of Korea; Department of Occupational and Environmental Medicine, Kangbuk Samsung Hospital, School of Medicine, Sungkyunkwan University, Seoul, Republic of Korea

**Keywords:** *N*,*N*-dimethylacetamide, occupational exposure, hepatotoxicity, liver injury, biological monitoring, occupational surveillance

## Abstract

**Objectives:**

*N*,*N*-Dimethylacetamide (DMAc) is a widely used industrial solvent and recognized occupational hepatotoxicant. Human evidence, however, appears inconsistent: clinically oriented reports describe overt liver injury in selected workers, whereas large epidemiologic studies often report null or nonspecific group-level biochemical findings. This review aimed to interpret these evidence streams with attention to latency, selective occurrence, recurrence after renewed exposure, and implications for occupational surveillance.

**Methods:**

We conducted a structured critical review based on a systematic PubMed/MEDLINE search through 22 May 2026. Human occupational studies were qualitatively compared with attention to latency, selective occurrence, clinical course after exposure modification, recurrence after renewed exposure, and exposure findings. Of 22 records, 15 underwent full assessment and 8 publications were included in the core synthesis.

**Results:**

Clinically oriented reports and cohorts of newly exposed workers repeatedly described DMAc-associated liver injury during the early exposure period, often within the first several weeks to months, affecting only a subset of exposed workers. Several reports described recurrence or worsening after renewed exposure. In contrast, epidemiologic studies in established worker populations primarily evaluated group-level liver enzyme outcomes and did not provide sufficient case-level information to assess latency, recurrence, or clinically overt hepatitis as a pattern-defined event.

**Conclusions:**

Published evidence remains limited and heterogeneous and does not establish DMAc-associated hepatotoxicity as a definitive idiosyncratic entity. Nevertheless, clinically apparent cases may require pattern-aware recognition, particularly during early exposure or renewed exposure. Population-level exposure control remains essential, but may be complemented by individual-level clinical vigilance.

## Teaser

**Key points**
 

- **What is already known on this topic:** DMAc is an occupational hepatotoxicant managed primarily through exposure control, biological monitoring, and liver function surveillance. Human studies have reported inconsistent findings, with clinically apparent cases described in some reports and null or nonspecific group-level findings in larger epidemiologic studies.
- **What this review adds:** This structured critical review interprets clinically oriented reports and epidemiologic surveillance studies as complementary evidence streams. Clinically apparent DMAc-associated liver injury was repeatedly described during the early exposure period, occurred selectively among exposed workers, and in selected cases recurred or worsened after renewed exposure. These features were not readily assessable in studies based primarily on group-level liver enzyme outcomes in established worker populations.
- **How this study might affect research, practice, or policy:** The findings do not establish a definitive idiosyncratic mechanism or permit quantitative risk estimation. However, they support a pattern-aware approach to occupational surveillance. Population-level exposure control remains essential, but early-phase clinical vigilance, timely exposure modification, and cautious decisions regarding renewed exposure may help identify uncommon clinically important cases.

## 1. Introduction

*N*,*N*-Dimethylacetamide (DMAc) is an organic solvent widely used, particularly in the production of synthetic fibers such as spandex and acrylic materials. Occupational exposure to DMAc has been associated with hepatotoxicity, and the liver has been consistently identified as the primary target organ of toxicity.^1^^,^^2^ DMAc hepatotoxicity has generally been managed within an exposure-control framework, reflecting evidence that higher exposure can increase hepatic risk. Experimental studies and occupational exposure assessments have suggested that higher levels of exposure are associated with an increased risk of hepatic injury.^1^ Based on this perspective, regulatory approaches have largely focused on exposure limits and environmental control measures to prevent toxicity.

Human occupational studies, however, have produced 2 apparently different evidence patterns. Cohort studies of newly exposed workers have reported liver injury or clinically apparent hepatitis occurring within a relatively short period after initial exposure, often affecting only a subset of workers despite similar exposure conditions.^3^^,^^4^ In contrast, large-scale epidemiologic studies conducted in established worker populations have not demonstrated a clear association between DMAc exposure and liver injury.^5–7^

These findings need not be inherently contradictory because the studies evaluated different populations and outcomes. Clinically oriented reports provide information on exposure-onset timing, symptoms, clinical course, and within-person recurrence, whereas studies based primarily on group-level liver enzyme outcomes are better suited to evaluating average biochemical effects in the observed workforce. However, the DMAc literature has not previously been synthesized specifically to determine whether these different study types collectively describe a recurring clinical phenotype or to clarify how such observations should be interpreted within an exposure-control framework.

These clinical features—including latency after first exposure, selective occurrence among exposed workers, incomplete individual-level predictability from exposure alone, and recurrence after renewed exposure—resemble patterns described in idiosyncratic drug-induced liver injury (IDILI).^8^^,^^9^ This resemblance raises a clinically relevant question but does not establish that DMAc-associated liver injury shares the immune, metabolic, or genetic mechanisms proposed for classical IDILI. In this review, the IDILI comparison is therefore used as a clinical pattern-recognition framework rather than as a mechanistic classification. Accordingly, this structured critical review aimed to compare clinically oriented and epidemiologic evidence on occupational DMAc-associated liver injury, with particular attention to latency after exposure initiation, selective occurrence among exposed workers, clinical course after exposure modification, and recurrence after renewed exposure. A further aim was to examine whether these evidence streams can be integrated into a clinically useful interpretive framework that complements, rather than replaces or relaxes, population-level exposure control.

## 2. Methods

### 2.1 Review design and analytic purpose

This study was designed as a structured critical review of published human studies on DMAc-associated liver injury in occupational settings. The aim of the review was to examine whether published clinical and epidemiologic evidence reveals a recurring clinical pattern—particularly with respect to latency after first exposure, selective occurrence among exposed workers, and recurrence after renewed exposure—that may be relevant to occupational surveillance and case interpretation. Evidence was identified through a systematic literature search and synthesized using a structured qualitative approach rather than quantitative pooling or meta-analysis. Pattern-based interpretation was used as a clinical and occupational health framework to compare the types of information provided by clinically oriented reports and group-level epidemiologic studies. This approach was not intended to estimate incidence, derive exposure–response thresholds, or establish a specific causal or mechanistic classification.

### 2.2 Literature search and evidence identification

A structured literature search was conducted to identify studies reporting liver injury associated with occupational exposure to DMAc. PubMed/MEDLINE was searched from database inception to 22 May 2026 using predefined combinations of DMAc-related terms, hepatic outcomes, and occupational context terms. No restrictions on publication year or language were applied at the search stage. The search was designed broadly enough to capture both clinically apparent liver injury and subclinical liver enzyme abnormalities relevant to DMAc-associated hepatotoxicity. A brief summary of the search strategy is provided here, and the full search syntax and screening summary are presented in Methods S1.

The updated search identified 22 records. After title and abstract screening, 15 records underwent full-text review or, when a conventional full text was not available, assessment of the most complete accessible publication record. All 15 records were assessed using the eligibility and evidence-classification criteria described below. Records that directly informed the primary review question were included in the core qualitative synthesis and detailed tabulation. Other potentially relevant records were retained for contextual interpretation, with their disposition and reasons for exclusion from the core synthesis documented in Methods S1.

### 2.3 Eligibility criteria and evidence classification

Eligible publications were primary human studies or reports involving occupational DMAc exposure and clinically apparent liver injury or liver biochemical outcomes. Publications were required to provide sufficient information on the occupational setting, exposure circumstances, study population, or hepatic outcome to permit interpretation in relation to the review question. Records were classified according to the type and granularity of evidence they provided, rather than according to whether their findings supported the proposed clinical pattern.

Studies reporting clinically apparent liver injury or well-defined occupational hepatic injury with sufficient information on timing, denominator, recurrence, or exposure context were summarized as clinically oriented evidence. Clinically oriented evidence was therefore defined as occupational human evidence providing sufficient case-level or cohort-level information to evaluate at least 1 pattern-relevant feature, including latency after exposure initiation, selective occurrence within an exposed population, clinical course following exposure modification, or recurrence after renewed exposure.

Studies that did not characterize clinically overt hepatitis but provided group-level liver enzyme outcomes in DMAc-exposed worker populations were classified separately as comparative epidemiologic evidence. This distinction was made to compare evidence streams that capture individual clinical events with those based primarily on biochemical surveillance outcomes at the population level.

Review articles, nonclinical studies, exposure-monitoring reports without human hepatic outcomes, reports of acute poisoning or substantially different exposure scenarios, and publications lacking sufficient occupational, clinical, or exposure information were not included in the core synthesis. Reports involving mixed chemical exposures were not treated as direct evidence of DMAc-specific causation; however, they could be considered contextually when they provided clinically informative observations, such as improvement following exposure cessation or recurrence after return to the same work environment.

### 2.4 Study selection process

All 15 records assessed in full were evaluated against the same eligibility and classification criteria. Eight publications directly contributed to either the clinically oriented pattern assessment or the comparative epidemiologic analysis and were therefore included in detailed tabulation. Publications providing information on latency, selective occurrence, recurrence after renewed exposure, denominator information, or exposure–outcome inconsistency are summarized in Table 1. Publications based primarily on group-level liver enzyme outcomes without sufficient characterization of clinically overt hepatitis, latency, or recurrence are summarized separately in Table 2 as comparative epidemiologic evidence.

**Table 1:** Clinically oriented occupational evidence with case-level temporal information on DMAc-associated liver injury.

Study	Setting, population, and evidence type	Latency/timing	Clinical events and recurrence	Exposure/biomonitoring findings and study limitations	Specific contribution to the review question
**Lee et al (2006)** ^3^	Korea; newly employed elastane fiber workers in DMAc-based wet spinning (*n* = 440)	Hepatic injury was concentrated within the first 1-2 months of employment; no cases were reported after more than 7 months of employment	28 cases among 440 workers (6.4%); recurrence after renewed exposure was not reported	Incidence differed across departments categorized by urinary *N*-methylacetamide levels. Individual exposure gradients and recurrence were not characterized in detail	Demonstrated concentration of hepatic injury during the early months of employment in a denominator-based cohort
**Jung et al (2007)** ^4^	Korea; newly exposed spandex fiber workers (*n* = 1045); cohort with clinical and biomonitoring information	Median latency was 47 days (range, 9-178 days); 76% of cases occurred within 2 months; no new cases were reported after more than 6 months of exposure	38 cases among 1045 workers (3.6%); recurrence occurred in 3 re-exposed workers, with shorter latency than the initial episode	Urinary *N*-methylacetamide levels were higher in departments where cases occurred, but liver injury developed in only a subset of exposed workers	Provided the clearest combined evidence on latency, selective occurrence, biomonitoring context, and earlier recurrence after re-exposure
**Wang and Chen (2020)^10^; Wang et al (2023)^a^** ^,^ ^11^	China; hospital-based clinical reports among spandex workers (*n* = 60); clinically ascertained toxic hepatitis cases	A population-based latency distribution was unavailable; exposure timing was assessed from individual occupational histories	The 2020 report described 60 hospital-based cases; the related 2023 report provided additional clinical and immunologic characterization. Recurrence or aggravation after renewed exposure was reported within this evidence stream	The source-population denominator and individual exposure gradients were unavailable; therefore, incidence and exposure–response relationships could not be estimated	Extended characterization of the clinical phenotype and recurrence, but without population-based incidence estimates
**Cheong et al (2007)^b^** ^,^ ^12^	Korea; synthetic fiber production with mixed solvent exposure, including DMAc and DMF (*n* = 48 exposed workers)	Timing after workplace exposure was described, but precise exposure-onset latency was not systematically reported	Five cases among 48 exposed workers (10.4%); recurrence occurred in 2 cases after return to work	Attribution to DMAc alone was uncertain because of mixed solvent exposure. The report was considered contextual evidence for recurrence after return to work rather than direct DMAc-specific evidence	Provided contextual within-person evidence of recurrence after return to the same work environment under mixed-solvent exposure

Abbreviations: DMAc, *N*,*N*-dimethylacetamide; DMF, dimethylformamide.

a The 2020 and 2023 reports were interpreted as a related hospital-based clinical evidence stream rather than as independent denominator-based cohort studies.

b This outbreak occurred under mixed solvent exposure conditions; attribution to DMAc alone is uncertain.

**Table 2:** Group-level epidemiologic evidence on biochemical liver outcomes in DMAc-exposed workers.

Study	Setting and analytic sample	Outcome approach	Main findings	Information available for clinical-pattern assessment	Specific contribution to the review question
**Spies et al (1995)** ^5^	Acrylic fiber production; established workers exposed to DMAc (*n* = 127) and referent workers (*n* = 217)	Group-level serum biochemical monitoring	No clinically overt cases were described among exposed workers, and no association was observed between DMAc exposure and liver enzyme levels	The study provided group-level biochemical comparisons in established workers, but case-level information on latency from exposure initiation, symptoms, exposure cessation, and recurrence was unavailable	Showed the absence of a clear group-level biochemical signal in established DMAc-exposed workers
**Antoniou et al (2021, 2022)** ^**a**^ ^,^ ^6^ ^,^ ^7^	Four European factories; 1844 ALT observations included in a retrospective occupational analysis	Analysis of ALT outcomes across occupational exposure metrics	Severe ALT elevations were uncommon, and no clear positive exposure–response relationship was reported across evaluated exposure metrics	The analyses provided group-level ALT findings but did not report sufficient case-level information on clinically apparent hepatitis, exposure-onset latency, clinical course after exposure modification, or recurrence	Provided group-level evidence across 1844 ALT observations without a consistent positive association across evaluated exposure metrics

Abbreviations: ALT, alanine aminotransferase; DMAc, *N*,*N*-dimethylacetamide.

a The 2021 and 2022 publications were based on the same underlying occupational data source and were interpreted as a single epidemiologic evidence stream.

Two publications based on the same underlying hospital-based case population were treated as a single evidence stream in interpretation.^10^^,^^11^ Two studies from 2021 and 2022 by Antoniou et al^6,7^ were likewise interpreted in relation to the same occupational data source, with the later publication^7^ considered informative for comparison with the primary report.^6^ A mixed-solvent outbreak investigation was retained in the main tabulated evidence because it provided clinically relevant information on recurrence after return to work, although attribution to DMAc alone was interpreted cautiously.^12^

The remaining records did not directly meet the informational requirements for the core synthesis. These included review-type articles without primary occupational data, reports of liver biochemical abnormalities or potential susceptibility markers without sufficient characterization of clinically apparent injury, reports involving acute or nonaligned exposure scenarios, mixed-exposure reports with insufficient DMAc-specific attribution, and publications lacking sufficient case-level or population-level detail for the primary comparison. These records were considered contextually where relevant but were not treated as core evidence. A screening summary and reasons for exclusion from the core synthesis are provided in Methods S1. One reviewer conducted the initial screening, eligibility assessment, evidence classification, and data extraction, and these processes and their results were subsequently verified by other members of the review team. Any discrepancies were resolved through discussion and consensus.

Because this review prioritized studies with sufficient detail to evaluate temporal pattern, selective occurrence, recurrence after renewed exposure, and clinically apparent disease, the resulting evidence base may overrepresent unusual, severe, or clinically recognized presentations. Accordingly, clinically oriented reports were used to characterize reported clinical patterns, not to estimate their frequency or representativeness among all DMAc-exposed workers. This potential selection and publication bias was considered explicitly when comparing clinically oriented evidence with group-level epidemiologic findings.

### 2.5 Data extraction and qualitative synthesis

Data were extracted on study design, country or region, industrial process, study population, latency or timing of onset, clinical events and available denominator information, recurrence after renewed exposure, and reported exposure–response patterns. For epidemiologic studies, information was additionally extracted on outcome definition and the extent to which clinically overt liver injury, latency, and recurrence could be assessed. When multiple reports originated from the same study population, they were integrated and interpreted as a single evidence stream.

Methodological strengths and limitations were evaluated qualitatively with particular attention to study design, case ascertainment, denominator availability, exposure characterization, outcome definition, temporal information, potential co-exposures, alternative causes of liver injury, and generalizability. Because the evidence consisted of heterogeneous case reports, outbreak investigations, worker cohorts, and group-level epidemiologic studies, a single quantitative risk-of-bias score was not considered appropriate.

Given the limited number of eligible core studies and the heterogeneity of study designs, outcome definitions, and exposure assessment methods, findings were synthesized qualitatively rather than quantitatively. The analysis focused on comparing clinically oriented reports with epidemiologic studies based primarily on group-level biochemical outcomes, and on examining how differences in study design, case ascertainment, and outcome definition may influence recognition of clinically apparent DMAc-associated liver injury in occupational settings. No pooled effect estimate, incidence estimate, or quantitative exposure–response analysis was attempted.

## 3. Results

### 3.1 Study selection and evidence classification

The updated search identified 22 records, of which 15 underwent full-text review or equivalent full-record assessment. Eight publications were included in detailed tabulation. Five publications contributed to the clinically oriented or contextual pattern assessment, representing 4 underlying evidence streams, and 3 publications contributed to the comparative epidemiologic assessment, representing 2 underlying evidence streams. Two publications based on the same hospital-based case population were treated as a single clinical evidence stream,^10^^,^^11^ and 2 epidemiologic publications based on the same underlying worker population were likewise interpreted together.^6^^,^^7^ Publications providing information on latency after first exposure, selective occurrence among exposed workers, recurrence after renewed exposure, denominator information, or exposure–outcome inconsistency are summarized as clinically oriented evidence in Table 1. These included cohort-based or case-oriented reports from newly exposed or clinically ascertained worker populations,^3^^,^^4^^,^^10^^,^^11^ as well as 1 mixed-solvent outbreak report that was considered contextual because it described recurrence after return to work, although attribution to DMAc alone was uncertain.^12^ Publications based primarily on group-level liver enzyme outcomes without sufficient characterization of clinically overt hepatitis, latency, or recurrence are summarized separately as comparative epidemiologic evidence in Table 2.^5–7^ Evidence classification was based on study design, outcome granularity, and the availability of temporal and case-level information rather than on the direction of the reported findings. The screening results and reasons for exclusion from the core synthesis are presented in Methods S1.

### 3.2 Clinically oriented evidence: latency and early-phase occurrence

Across the clinically oriented reports, DMAc-associated liver injury was repeatedly described during the early period after initial occupational exposure. In the cohort of newly employed elastane fiber workers reported by Lee et al,^3^ hepatic injury occurred predominantly within the first 1-2 months of employment, and no new cases were reported after more than 7 months of employment. In the larger Korean spandex cohort reported by Jung et al,^4^ the median latency to onset was 47 days, with most cases occurring within 2 months and no new cases reported after more than 6 months of exposure.

The hospital-based Chinese case series by Wang and Chen^10^ did not provide a denominator-based latency distribution comparable to the Korean cohorts, but the cases were clinically ascertained as DMAc-induced toxic hepatitis among spandex workers. Wang et al,^11^ who further characterized a related hospital-based case population, provided additional clinical and immunologic observations but these were interpreted as part of the same broad evidence stream rather than as an independent denominator-based cohort.

Thus, in the 2 denominator-based cohorts of newly exposed workers, reported hepatic injury was concentrated within the first several weeks to months after exposure initiation. Comparable latency distributions could not be derived from the hospital-based case series.

### 3.3 Selective occurrence among exposed workers

Clinically oriented reports also described selective occurrence of liver injury among exposed workers. In the cohort reported by Lee et al,^3^ 28 cases of DMAc-induced hepatic injury were identified among 440 newly employed workers. In the larger cohort reported by Jung et al,^4^ 38 cases were identified among 1045 DMAc-exposed workers. In the hospital-based series reported by Wang and Chen,^10^ 60 patients with DMAc-induced toxic hepatitis were described, although the source population denominator was unavailable.

In both denominator-based cohorts, hepatic injury was reported in a minority of the exposed population rather than uniformly among workers in the same industrial setting. The available studies did not determine the relative contributions of individual exposure variability, host-related factors, or other workplace conditions to this selective occurrence.

### 3.4 Exposure level and incomplete individual-level predictability

Several reports provided evidence that higher exposure or higher biomonitoring levels may increase risk at the group level. Lee et al^3^ reported higher incidence across departments classified by urinary *N*-methylacetamide levels. Jung et al^4^ similarly reported higher urinary *N*-methylacetamide distributions in departments where cases occurred than in departments without cases. These findings demonstrated department-level relationships between exposure indicators and hepatic outcomes.

However, the available clinical reports did not provide sufficient evidence to define a precise individual-level exposure threshold that reliably predicted which workers would develop clinically apparent liver injury. The denominator-based studies reported group-level or department-level exposure differences, but liver injury developed in only a subset of exposed workers.^4^ In Wang and Chen,^10^ exposure–response relationships could not be assessed because the cases were hospital-based and the denominator of exposed workers was not available.

Overall, the available evidence supported exposure-related differences at the group or departmental level but was insufficient to characterize a reliable individual-level threshold for clinically apparent injury.

### 3.5 Recurrence or worsening after renewed exposure

Recurrence after renewed exposure was one of the most clinically informative features in the available evidence. In the Korean spandex cohort, recurrence was reported in 3 workers after re-exposure, with shorter latency than during the initial episode.^4^ In the hospital-based evidence stream, recurrence or aggravation after renewed exposure was also described, although the denominator and exposure context were not comparable to those of the Korean occupational cohorts.^10^^,^^11^ Cheong et al^12^ described recurrence after return to work in a mixed-solvent outbreak investigation, but attribution to DMAc alone was uncertain because workers were exposed to multiple chemicals, including DMAc and other hepatotoxic solvents.

Recurrence was therefore documented in a small number of workers across heterogeneous exposure settings. The available reports did not establish its frequency among all previously affected workers or distinguish among potential biological mechanisms.

### 3.6 Comparative epidemiologic evidence based on group-level biochemical outcomes

In contrast to the clinically oriented reports, comparative epidemiologic studies primarily evaluated group-level liver enzyme outcomes in DMAc-exposed worker populations. Spies et al^5^ investigated established acrylic fiber workers exposed to DMAc and referent workers using serum biochemical tests. In that study, no clinically overt cases were reported among 127 exposed workers, and no association between DMAc exposure and liver enzyme levels was observed.

Antoniou et al^6^ conducted a large retrospective analysis of workers from 4 European factories based on 1844 alanine aminotransferase (ALT) observations and did not identify a clear positive association between DMAc exposure metrics and liver toxicity. The related publication by Antoniou et al^7^ was interpreted in relation to the same underlying occupational data source and was considered informative for the consistency of the null group-level findings. In these studies, severe ALT elevations were uncommon, and the available data did not provide a denominator-based characterization of clinically overt hepatitis with latency, recurrence, or exposure-modification patterns.

These studies provided evidence regarding average or group-level biochemical outcomes in established worker populations. However, the reported data did not include sufficient case-level information to characterize exposure-onset latency, symptom course, improvement after exposure cessation, or recurrence after renewed exposure.

### 3.7 Integrated comparison of evidence streams

Across the clinically oriented evidence, reported hepatic injury was concentrated during the initial months of exposure in 2 newly exposed worker cohorts, occurred in a minority of exposed workers, and recurred or worsened after renewed exposure in a small number of cases.^3^^,^^4^^,^^10–12^ Exposure and biomonitoring findings indicated group-level or departmental exposure relationships, but the available studies did not define a reliable individual-level threshold for clinically apparent injury. By contrast, comparative epidemiologic studies of established workers reported no clear group-level biochemical excess or positive exposure–response relationship and did not provide the longitudinal case-level information required to evaluate latency, clinical course, or recurrence.^5–7^ The 2 evidence groups therefore differed not only in their findings but also in worker population, outcome definition, and the availability of temporal clinical information. The practical implications of this interpretation are summarized in (Figure 1).

**Figure 1:**
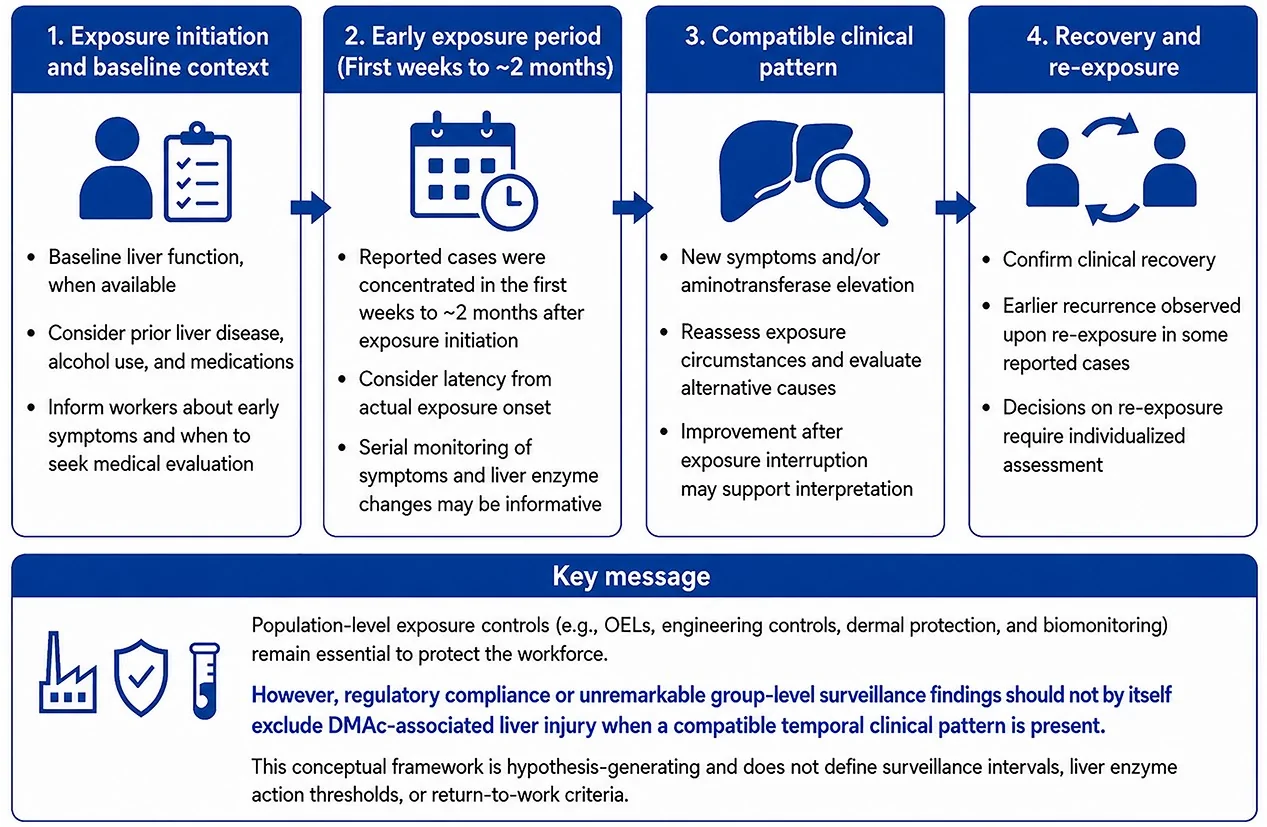
Conceptual framework for recognizing and interpreting clinically apparent *N*,*N*-dimethylacetamide (DMAc)-associated liver injury. The figure summarizes recurring temporal and clinical features reported in occupational studies of DMAc-associated liver injury. Population-level exposure controls, including occupational exposure limits, engineering controls, reduction of airborne and dermal exposure, personal protective equipment, and biological monitoring, remain the foundation of prevention. The framework highlights the first weeks to approximately 2 months after exposure initiation, serial monitoring of symptoms and liver enzyme changes, evaluation of alternative causes, improvement after exposure interruption, and earlier recurrence after re-exposure in some reported cases. It is intended as a conceptual, hypothesis-generating aid for interpreting uncommon clinically apparent cases and does not define evidence-based surveillance intervals, liver enzyme action thresholds, or return-to-work criteria.

## 4. Discussion

### 4.1 Principal interpretation: two complementary evidence streams

The main interpretation of this review is that clinically oriented reports and group-level epidemiologic studies should be understood as complementary evidence streams rather than as simply conflicting findings. Clinically oriented reports described DMAc-associated liver injury with features relevant to case recognition, including occurrence during an early exposure period, selective development among exposed workers, incomplete individual-level predictability from exposure information alone, and recurrence or worsening after renewed exposure.^3^^,^^4^^,^^10^^,^^11^ In contrast, larger epidemiologic studies in established worker populations primarily evaluated group-level biochemical outcomes and generally did not provide sufficient case-level information to assess latency, recurrence, or clinically overt hepatitis as a pattern-defined event.^5–7^

The principal contribution of the present review is therefore not the estimation of quantitative risk, but the integration of clinically oriented and epidemiologic evidence into a coherent interpretive framework. Across independent reports, clinically apparent DMAc-associated liver injury showed a recurring pattern characterized by delayed onset after exposure initiation, selective occurrence among exposed workers, improvement after exposure cessation, and occasional recurrence after renewed exposure, whereas these case-level features were generally not evaluated in studies based primarily on group-level biochemical outcomes.

This interpretation does not establish DMAc-associated hepatotoxicity as a definitive idiosyncratic entity, nor does it permit estimation of incidence, prevalence, or quantitative risk. The number of clinically detailed reports is small, and the available evidence is heterogeneous. Furthermore, the clinically oriented evidence synthesized in this review may be subject to selection and publication biases. Clinically apparent, severe, recurrent, or otherwise unusual cases are more likely to undergo clinical evaluation and be reported in the literature than mild, transient, or subclinical liver enzyme abnormalities. Consequently, the clinical patterns identified in this review should not be interpreted as representing the full spectrum or frequency of DMAc-associated liver effects among exposed workers, but rather as recurring observations from the subset of clinically recognized cases available in the published literature. Nevertheless, the recurring clinical observations are occupationally relevant because they suggest that uncommon clinically apparent cases may require a different interpretive approach from that used in routine group-level biochemical surveillance. Accordingly, the value of the present review lies not in estimating the frequency or quantitative risk of DMAc-associated liver injury, but in clarifying how clinically apparent cases may be recognized and interpreted when they occur. This interpretive framework should therefore be regarded as hypothesis-generating rather than as evidence establishing causality or defining surveillance practice.

### 4.2 Why clinically apparent cases may be less visible in routine surveillance

The contrast between clinically oriented reports and large epidemiologic studies has practical implications for occupational surveillance. In the clinically oriented literature, cases were described after an initial exposure period, in only a subset of exposed workers, and in some instances recurred after return to exposure.^3^^,^^4^^,^^10^^,^^11^ By contrast, the epidemiologic studies were based primarily on group-level liver enzyme monitoring in established worker populations and did not directly assess latency, recurrence, or clinically overt hepatitis.^5–7^

Several complementary explanations may account for this apparent discrepancy. First, clinically apparent liver injury may genuinely be uncommon under contemporary occupational conditions with improved exposure control.^1^^,^^2^ Second, early-phase events may be preferentially detected in cohorts of newly exposed workers, whereas studies conducted primarily among established workers may underrepresent such events because affected workers discontinue exposure, transfer to other tasks, or leave employment before long-term surveillance, consistent with a possible healthy worker survivor effect. Third, historical differences in workplace exposure levels, engineering controls, industrial processes, and worker training between earlier and more recent studies may also have influenced the frequency and recognition of clinically apparent liver injury. Finally, epidemiologic studies based primarily on group-level liver enzyme distributions are inherently less suited to evaluating temporally patterned clinical phenomena, including latency after first exposure, recurrence after renewed exposure, and clinically overt hepatitis as an individual clinical event. These explanations are not mutually exclusive, and the currently available evidence does not permit estimation of their relative contributions.

Accordingly, the present review does not interpret epidemiologic null findings as contradicting the clinically oriented literature. Rather, the available evidence suggests that different study designs capture different dimensions of DMAc-associated liver injury. Clinically oriented investigations may be more informative for recognizing uncommon, temporally patterned clinical events, whereas surveillance-based epidemiologic studies remain essential for evaluating average biochemical outcomes and the effectiveness of population-level exposure control. Therefore, the observed discrepancy should be interpreted as complementary evidence rather than conflicting evidence. This interpretation is intended as a conceptual framework for understanding the current literature and should not be regarded as evidence supporting specific surveillance intervals or occupational management recommendations. Importantly, these observations should not be interpreted as evidence that early-occurring clinically apparent liver injury is explained predominantly by individual susceptibility, because several workplace-related and epidemiologic factors may contribute simultaneously to the observed pattern. Although the relative contribution of these explanations cannot be determined from the available evidence, they provide a plausible explanation for why clinically oriented reports and established-worker epidemiologic studies may describe different but not necessarily contradictory aspects of DMAc-associated liver injury.

### 4.3 Exposure control and the complementary role of clinical recognition

The findings of this review should not be interpreted as challenging the importance of exposure control or occupational exposure limits. DMAc hepatotoxicity remains an exposure-related occupational hazard, and higher exposure or higher biomonitoring levels may increase hepatic risk at the group level. The cohort findings summarized in this review similarly indicate that departments or worker groups with higher urinary *N*-methylacetamide levels may experience greater hepatic risk.^3^^,^^4^ Nothing in the present review supports replacing, relaxing, or deprioritizing population-level exposure control. Occupational exposure limits, engineering controls, dermal exposure reduction, biological monitoring, and worker education remain the foundation of DMAc risk management.^1^^,^^2^ The principal implication of this review concerns the interpretation of uncommon clinically apparent events after exposure has occurred. Exposure measurements and routine surveillance remain essential, but they may be complemented by clinical information including exposure timing, serial liver enzyme changes, symptoms, improvement after exposure cessation, and any previous response to similar exposure.^3^^,^^4^^,^^10^^,^^11^ The available evidence does not support defining specific monitoring intervals, liver enzyme action thresholds, or universal return-to-work criteria. Rather, the principal contribution of the present review is to provide a clinical interpretive framework for evaluating uncommon clinically apparent liver injury after DMAc exposure. From an occupational health perspective, effective exposure control and recognition of compatible clinical patterns should be regarded as complementary rather than competing strategies. Although population-level exposure reduction remains the cornerstone of prevention, compliance with current exposure standards or routine surveillance findings should not automatically preclude consideration of DMAc-associated liver injury when a compatible temporal clinical pattern is present. Clinical judgment should therefore integrate exposure history, exposure timing, serial liver enzyme changes, symptoms, improvement after exposure cessation, and any previous response to similar exposure, together with workplace-specific exposure assessment and applicable occupational health regulations.

### 4.4 Mechanistic interpretation and biological plausibility

The recurring clinical pattern identified in this review should not be interpreted as evidence of a specific biological mechanism. Although the observed latency, selective occurrence, and recurrence after renewed exposure resemble features described in IDILI, the currently available evidence does not distinguish among direct metabolic toxicity, host metabolic variation, immune involvement, or combinations of these processes.^8^^,^^9^

DMAc undergoes oxidative metabolism, with *N*-methylacetamide serving as a major urinary metabolite used in biological monitoring. Other identified urinary products include *N*-hydroxymethyl-*N*-methylacetamide and *S*-(acetamidomethyl) mercapturic acid.^1^^,^^2^^,^^13^ Experimental liver microsomal studies have implicated cytochrome P450, probably CYP2E1, and have demonstrated the formation of free-radical intermediates during DMAc metabolism.^14^ Although these findings provide a biologically plausible context for hepatotoxicity, they do not explain why clinically apparent liver injury develops only in a subset of exposed workers after a characteristic latency period. Limited clinical observations have also suggested possible host-related factors. Changes in circulating cytokines paralleled liver injury in one case series, and catalase (CAT) gene polymorphisms were associated with abnormal liver function in another study.^11^^,^^15^ However, neither finding establishes an immune-mediated mechanism or provides a validated marker of individual susceptibility. These observations should therefore be regarded as preliminary biological clues rather than mechanistic evidence.

Recurrence or more rapid worsening after renewed exposure provides clinically relevant within-person evidence that response to DMAc may vary among workers.^4^^,^^10^^,^^11^ Nevertheless, the small number of recurrent cases does not establish immune sensitization, metabolic adaptation, or any other specific biological mechanism. Overall, the present review neither proposes susceptibility-based screening nor infers a defined genetic, metabolic, or immunologic pathway. The more defensible implication remains clinical: when liver injury develops after a temporally plausible exposure period and improves following exposure cessation, renewed exposure should be approached cautiously while mechanistic uncertainty is explicitly acknowledged.

### 4.5 DMAc in the broader context of occupational solvent hepatotoxicity

DMAc should also be interpreted within the broader context of occupational solvent-induced liver injury. Among structurally related solvents, dimethylformamide (DMF) has long been recognized as a cause of occupational hepatotoxicity. Although reported clinical presentations vary, delayed onset after exposure initiation, selective occurrence among exposed workers, and occasional recurrence after renewed exposure have all been described.^1^^,^^16–18^ These observations suggest that temporally patterned liver injury is not unique to DMAc, although important differences in metabolism, exposure characteristics, and evidence quality preclude assuming a shared biological mechanism.

Trichloroethylene (TCE) represents a different but informative comparison. Unlike DMAc, TCE-associated hypersensitivity syndrome is characterized by systemic manifestations, including rash, fever, eosinophilia, and lymphadenopathy, and is supported by stronger immunogenetic evidence such as the association with HLA-B*13:01.^19–21^ Accordingly, TCE illustrates a more clearly established susceptibility-related phenotype, whereas the biological basis of DMAc-associated liver injury remains considerably less well defined. Taken together, these comparisons broaden the perspective from a single solvent to occupational solvent-related hepatotoxicity more generally. They suggest that delayed, selective, and occasionally recurrent liver injury may represent a clinically relevant pattern across several occupational solvents, although the underlying biological mechanisms and strength of evidence differ substantially among individual agents. Rather than implying a common mechanism, these comparisons highlight the importance of recognizing recurrent clinical phenotypes while interpreting each solvent according to its own toxicological and epidemiologic evidence. From this perspective, DMAc should not be viewed as an isolated occupational hepatotoxin but as part of a broader spectrum of solvent-associated liver injury in which recurring clinical patterns may provide complementary information beyond conventional population-based surveillance.

### 4.6 Practical implications for early recognition and exposure management

Figure 1 summarizes a conceptual and hypothesis-generating framework for interpreting clinically apparent DMAc-associated liver injury. It is not intended to prescribe surveillance intervals, liver enzyme action thresholds, exposure-removal criteria, or return-to-work decisions. Rather, it identifies clinical circumstances in which closer attention may be warranted, including the initiation of DMAc exposure, entry into a new or modified process, and consideration of renewed exposure after a previous liver injury episode. The term “early exposure period” should be understood descriptively rather than as a validated surveillance window. In the 2 newly exposed worker cohorts, most cases occurred during the first 1-2 months after exposure initiation; the reported latency ranged from 9 to 178 days, and no new cases were identified after approximately 6-7 months of exposure.^3^^,^^4^ These findings suggest that the first weeks to months after actual exposure initiation may be particularly relevant for clinical recognition.

However, the limited and historically specific evidence does not establish a fixed time boundary beyond which clinically apparent injury cannot occur. Where liver function surveillance is required by regulation or workplace policy, its timing may be more clinically informative when referenced to actual DMAc exposure onset rather than employment date alone. Baseline assessment before or near exposure initiation may facilitate interpretation of subsequent changes. Additional clinical attention may also be appropriate after a substantial process change, an unexpected increase in exposure, or renewed exposure following a previous hepatic event. Nevertheless, the available studies do not provide sufficient evidence to recommend a specific testing interval, and surveillance frequency should remain determined by applicable regulations, workplace-specific exposure assessment, and occupational health judgment. Although these observations do not define a universal surveillance window, they identify a period of heightened clinical relevance that was consistently observed across the available newly exposed worker cohorts.

The clinical significance of liver enzyme elevation should likewise be interpreted in context. Serial liver enzyme changes interpreted together with exposure timing and clinical symptoms appear substantially more informative than isolated biochemical abnormalities alone. A single mild or transient ALT or aspartate aminotransferase (AST) elevation is nonspecific and does not by itself establish DMAc-associated liver injury. Concern may increase when a newly developed, persistent, or progressive aminotransferase elevation occurs after a temporally plausible exposure period, particularly when accompanied by compatible symptoms, corroborating exposure information, improvement after exposure cessation, or recurrence following renewed exposure.^3^^,^^4^^,^^10^^,^^11^ Alternative hepatic causes should be evaluated concurrently, and exposure interruption or modification may be considered while the clinical assessment is ongoing. These features may strengthen case-level suspicion, but they do not establish causality without appropriate differential evaluation.

Re-exposure after recovery should be considered cautiously because recurrence or more rapid worsening has been described in a small number of workers.^4^^,^^10^^,^^12^ However, the evidence is insufficient to define universal return-to-work criteria or to conclude that re-exposure will invariably result in recurrence. Decisions regarding return to similar work should therefore be individualized on the basis of the certainty and severity of the previous hepatic event, the availability of alternative explanations, current workplace exposure conditions, and the feasibility of exposure elimination or substantial reduction.

### 4.7 Strengths and limitations

A strength of this review is its structured comparison of evidence streams that address different aspects of DMAc-associated hepatotoxicity. The literature was identified through a systematic search, and publications were classified according to predefined analytic roles rather than pooled across clinically dissimilar outcomes. Clinically oriented reports were used to examine latency, selective occurrence, exposure modification, and recurrence, whereas epidemiologic studies were evaluated for their contribution to group-level biochemical and exposure–response interpretation.^3–7^^,^^10–12^ This approach made it possible to examine why uncommon clinically recognized events and largely null population-level findings may coexist without assuming that either evidence stream is inherently more valid.

Several limitations should nevertheless be emphasized. First, the number of clinically informative human studies was small, and the available reports were heterogeneous in study design, worker population, denominator availability, exposure characterization, case definition, and outcome assessment. Second, the pattern-based synthesis necessarily prioritized publications containing sufficient case-level information to evaluate timing, clinical course, and renewed exposure. This selection strategy improves the ability to examine the review question but may preferentially capture unusual, severe, recurrent, or clinically recognized presentations. Such cases may also be more likely to be investigated and published than mild, transient, asymptomatic, or uncomplicated liver enzyme abnormalities. The identified clinical pattern should therefore not be interpreted as representing the full clinical spectrum or frequency of liver effects among all DMAc-exposed workers.

Third, clinically oriented and epidemiologic studies did not assess identical outcomes and cannot be compared as if they represented equivalent study designs. The hospital-based case series lacked source-population denominators and sufficiently detailed individual exposure gradients, whereas some cohort and surveillance studies did not provide case-level information on latency, symptoms, exposure cessation, or recurrence.^5–7^^,^^10^^,^^11^ Mixed-exposure observations could not establish attribution to DMAc alone and were therefore interpreted only as contextual evidence.^12^ Differences in historical exposure intensity, industrial processes, exposure controls, worker education, and workforce retention further limit the extent to which findings from older reports can be generalized to contemporary workplaces.

Fourth, mechanistic evidence remains preliminary. Experimental metabolic findings, cytokine changes, recurrence after renewed exposure, and reported host-related modifiers provide possible biological context but do not establish a unified metabolic, immune-mediated, or genetic mechanism.^11^^,^^13–15^ In particular, the small number of recurrent cases cannot determine whether recurrence reflects immune sensitization, metabolic variation, another form of individual susceptibility, or continued exposure-related toxicity.

Finally, the small and heterogeneous evidence base does not permit estimation of current incidence, prevalence, individual dose–response thresholds, or the effectiveness of particular surveillance intervals. It also does not support validated liver enzyme action thresholds, susceptibility-based screening, or universal return-to-work criteria. Accordingly, this review should be interpreted as a cautious and hypothesis-generating critical synthesis rather than as evidence establishing causality or a definitive idiosyncratic hepatotoxicity entity. Its principal contribution is to clarify how recurring clinical observations may be interpreted without overstating their frequency, mechanism, or quantitative implications. Despite these limitations, the structured comparison presented here clarifies how recurring clinical observations can coexist with largely unremarkable findings from established-worker epidemiologic studies and identifies priorities for future prospective research.

## 5. Conclusions

Published occupational evidence on DMAc-associated liver injury remains limited and heterogeneous. Nevertheless, clinically oriented reports describe a recurring pattern in which clinically apparent liver injury developed after an initial exposure period, affected only a subset of exposed workers, and occasionally recurred or worsened after renewed exposure.^3^^,^^4^^,^^10^^,^^11^ By contrast, studies of established worker populations generally found no clear group-level biochemical excess and provided limited case-level information on latency, clinical course, or recurrence.^5–7^ These 2 evidence streams should therefore be interpreted as complementary rather than contradictory.

Population-level prevention through occupational exposure limits, engineering controls, reduction of airborne and dermal exposure, appropriate protective measures, and biological monitoring remains the foundation of DMAc risk management.^1^^,^^2^ However, effective exposure control or compliance with current exposure standards should not automatically exclude consideration of DMAc-associated liver injury when a compatible temporal and clinical pattern is present. Exposure timing, serial liver enzyme changes, symptoms, improvement after exposure cessation, and any previous response to similar exposure may provide clinically relevant information beyond isolated exposure measurements or group-level surveillance findings.

The principal contribution of this review is not the definition of a new exposure threshold or a specific biological mechanism, but the identification of a recurring, temporally patterned clinical phenotype across independent occupational reports. The convergence of delayed onset, selective occurrence, improvement after exposure cessation, and occasional recurrence after renewed exposure is not readily explained solely by incidental liver enzyme variation or by the design of group-level surveillance studies. This pattern-aware framework remains conceptual and hypothesis-generating and does not establish a specific idiosyncratic, immune-mediated, metabolic, or genetic mechanism, nor does it permit quantitative risk estimation or define universal surveillance and return-to-work criteria. Prospective studies of newly exposed workers, incorporating individual exposure assessment, serial clinical evaluation, and longitudinal follow-up, are needed to determine the frequency, determinants, and contemporary relevance of this clinical phenotype.

## Supplementary Material

260721_Supplementary_Final_uiag043

## Data Availability

No new datasets were generated or analyzed in this study. All data discussed in this review are available in the published articles cited in the reference list.
