# Supplementary material for: Dimethylacetamide-associated liver injury in workers: clinical patterns of latency, selective occurrence, and implications for occupational surveillance
Source: J Occup Health. 2026 Sep 28;68(1):uiag043. doi: 10.1093/joccuh/uiag043 (PMC13728918; doi:10.1093/joccuh/uiag043)
Supplement: Supplementary file 1 [file 260721_supplementary_final_uiag043.docx]

**Supplementary Methods S1. Literature Search Strategy**

**Databases**
PubMed/MEDLINE

**Search period**
From database inception to **22 May 2026**.

No restrictions on publication year or language were applied at the search stage.

**Search Strategy (PubMed)**

The following search strategy was used to identify human studies evaluating occupational exposure to N,N-dimethylacetamide (DMAc) and hepatic outcomes.

(dimethylacetamide[tiab] OR "N,N-dimethylacetamide"[tiab] OR DMAC[tiab] OR DMAc[tiab] OR "dimethyl acetamide"[tiab] OR "N-methylacetamide"[tiab] OR NMAc[tiab] OR "127-19-5"[rn]) AND (liver[tiab] OR hepatic[tiab] OR hepatocellular[tiab] OR hepatotoxic*[tiab] OR "liver injury"[tiab] OR "liver dysfunction"[tiab] OR hepatitis[tiab] OR ALT[tiab] OR AST[tiab] OR transaminase*[tiab] OR "liver enzyme"[tiab]) AND (worker*[tiab] OR workplace*[tiab] OR occupational[tiab] OR factory[tiab] OR factories[tiab] OR spandex[tiab] OR elastane[tiab] OR fiber[tiab] OR fibre[tiab] OR textile*[tiab]) AND Humans[Mesh]

**Notes on search strategy**

The search was restricted primarily to Title/Abstract fields to improve specificity for occupational and clinical relevance. Chemical names, abbreviations, and the registry number for DMAc (CAS RN: 127-19-5) were included to increase retrieval of relevant records. Hepatic outcome terms were defined broadly to capture both clinically apparent liver injury and subclinical liver enzyme abnormalities. Occupational context terms were included to preferentially identify workplace exposure settings, particularly those relevant to synthetic fiber manufacturing. The Humans[Mesh] term was applied to focus retrieval on human evidence.

The search was designed to identify both clinically oriented reports and epidemiologic studies with group-level biochemical outcomes. The purpose of the search was to provide a transparent and reproducible evidence base for qualitative comparison rather than quantitative pooling or estimation of incidence, effect size, or exposure–response thresholds.

**Screening Summary**

Records identified through PubMed/MEDLINE: 22

Records screened by title/abstract: 22

Records excluded after title and abstract screening: 7

Records undergoing full-text or equivalent assessment: 15

Records used for detailed tabulation: 8 publications

-Clinically oriented or contextual evidence: 5 publications representing 4 evidence streams

-Comparative epidemiologic evidence: 3 publications representing 2 evidence streams

*Two publications derived from the same hospital-based clinical population were interpreted as one clinical evidence stream. Two epidemiologic publications based on the same underlying occupational data source were likewise interpreted as one comparative evidence stream.

Records not included in detailed tabulation: 7

Clinically oriented and comparative epidemiologic evidence are summarized in Tables 1 and 2, respectively; contextual evidence is described in the text where relevant.

**Reasons for exclusion from detailed tabulation**

Records were not included in detailed tabulation when they did not directly contribute sufficient information to the primary comparison between clinically oriented and group-level epidemiologic evidence. These included review articles without primary occupational human data, nonclinical or exposure-monitoring studies without human hepatic outcomes, reports involving acute poisoning or exposure scenarios not comparable with routine occupational DMAc exposure, mixed-exposure reports with insufficient basis for DMAc-specific attribution, studies of biochemical abnormalities or potential susceptibility markers without adequate characterization of clinically apparent liver injury, and publications lacking sufficient clinical, temporal, or exposure detail to evaluate latency, clinical course, recurrence, or population-level outcomes. Such records were considered contextually where relevant but were not treated as core evidence for the pattern-based synthesis.
